# Fiber-Laser-Based Ultrasound Sensor for Photoacoustic Imaging

**DOI:** 10.1038/srep40849

**Published:** 2017-01-18

**Authors:** Yizhi Liang, Long Jin, Lidai Wang, Xue Bai, Linghao Cheng, Bai-Ou Guan

**Affiliations:** 1 Guangdong Provincial Key Laboratory of Optical Fiber Sensing and Communications, Institute of Photonics Technology, Jinan University, Guangzhou 510632, China; 2 Department of Mechanical and Biomedical Engineering, City University of Hong Kong, 83 Tat Chee Ave, Kowloon, Hong Kong SAR, China

## Abstract

Photoacoustic imaging, especially for intravascular and endoscopic applications, requires ultrasound probes with miniature size and high sensitivity. In this paper, we present a new photoacoustic sensor based on a small-sized fiber laser. Incident ultrasound waves exert pressures on the optical fiber laser and induce harmonic vibrations of the fiber, which is detected by the frequency shift of the beating signal between the two orthogonal polarization modes in the fiber laser. This ultrasound sensor presents a noise-equivalent pressure of 40 Pa over a 50-MHz bandwidth. We demonstrate this new ultrasound sensor on an optical-resolution photoacoustic microscope. The axial and lateral resolutions are 48 μm and 3.3 μm. The field of view is up to 1.57 mm^2^. The sensor exhibits strong resistance to environmental perturbations, such as temperature changes, due to common-mode cancellation between the two orthogonal modes. The present fiber laser ultrasound sensor offers a new tool for all-optical photoacoustic imaging.

Photoacoustic tomography (PAT) is a phenomenally growing imaging technology which offers high-contrast, high-resolution and non-invasive imaging in deep tissue^1^,^2^. PAT has received great interests due to promising applications in disease diagnoses and life science researches^3^,^4^,^5^,^6^,^7^. PAT detects optically induced ultrasound signals to form three-dimensional images^8^,^9^,^10^. Further development of PAT demands high-performance ultrasound sensors with high sensitivity, small size, broad bandwidth and great stability. Specifically, intravascular and endoscopic photoacoustic imaging requires ultrasound probes with miniature sizes and high sensitivity^11^. However, piezoelectric detectors are limited by the tradeoff between sensor size and sensitivity. For instance, a polyvinylidene (PVDF) needle hydrophone with a diameter of 75 μm presents much lower sensitivity than that with ordinary size, with a noise-equivalent pressure (NEP) of 6 kPa over 100 MHz^12^. In recent years, a number of photonic ultrasound sensors have been developed with outstanding sensitivity, broad bandwidth and compact size^13^,^14^,^15^, optical transparency, and even possibility for non-contact measurement^16^,^17^,^18^. Photonic ultrasound sensors usually employ high-finesse optical resonators to detect incident elastic waves. For instance, a polymer micro-ring resonator (60 μm in diameter) has presented 105 Pa NEP over a bandwidth of 350 MHz^19^,^20^. Ultrasound transducers based on planar Fabry-Perot (FP) cavity have reached a NEP of 210 Pa over 20 MHz bandwidth^21^. A reflection-mode PAM system using FP etalons for optical ultrasound detection has been developed^22^,^23^, which has achieved a NEP of 80 Pa and bandwidth of 18 MHz. The high sensitivity, however, raises its susceptibility to environmental disturbances. Complicated stabilization strategies have to be employed to enhance the resistance to external perturbations.

Advanced fiber laser techniques have been developed for optical communication, sensing, measurement, defense and industrial applications. Optical fibers with lengths from millimeters to centimeters have been exploited as photonic sensors for the detection of weak strains and related mechanical parameters^24^,^25^,^26^,^27^. Fiber laser sensors exhibit extraordinary performances in the detection of low-frequency (typically lower than ~2 kHz) acoustic waves, taking advantage of extremely narrow linewidth, sensitization packaging and phase demodulation methods. However, the bandwidth of most fiber-laser-based acoustic sensors has been limited to below ~1 MHz by the slow speed of phase-generated carrier (PGC) demodulation technique, as well as the relaxation oscillations of rare-earth doped fibers. The detection of a spherical-wave photoacoustic signal using existing fiber-laser sensor is challenging due to the destructive wavefront integration along the fiber length.

In this work, we present the development of a new fiber-laser-based photoacoustic detector. The laser emits two orthogonal polarization modes with slightly different frequencies, yielding a radio-frequency beat signal. Ultrasound waves induce birefringence changes, resulting in beat-frequency variation. This ultrasound sensor presents a NEP of 40 Pa over a 50 MHz bandwidth. The detector exhibits strong resistance to environmental perturbations due to common-mode cancellation between the two orthogonal modes, which is beneficial for practical imaging applications. We demonstrate photoacoustic imaging capability with this ultrasound sensor. The axial and lateral resolutions are 48 μm and 3.3 μm, respectively. The field-of-view (FOV) is up to 1.57 mm^2^. The fiber-laser ultrasound detector offers a valuable tool for all-optical photoacoustic imaging techniques.

## Results

### Working principle

**Figure 1:**
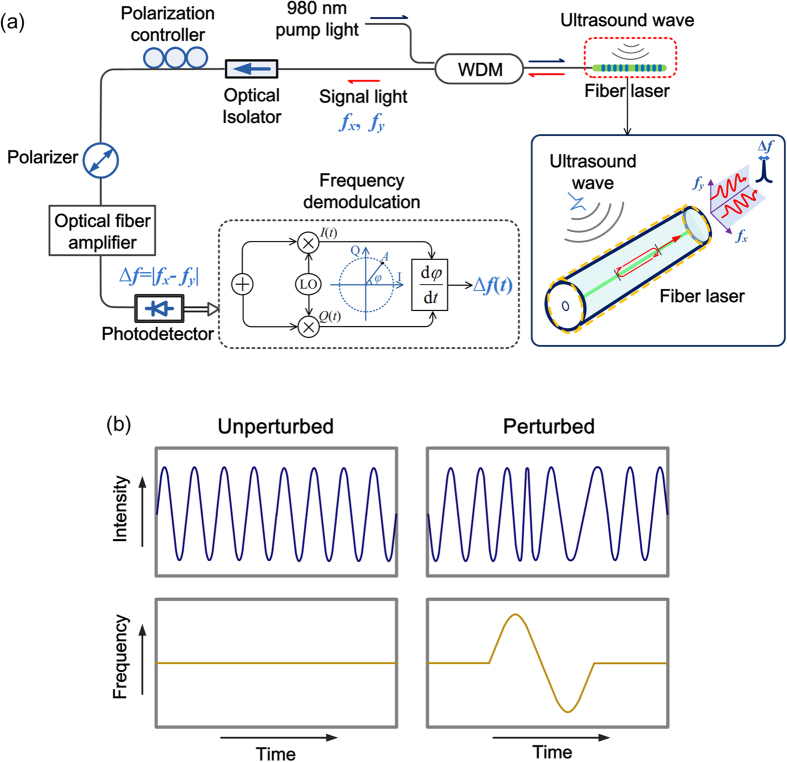
(**a**) Schematic of ultrasound detection with a fiber laser. WDM: Wavelength-division multiplexer; LO: Local oscillator. (**b**) Schematic of the acoustically induced frequency variation of the output beat signal Δf(t).

**Figure 2:**
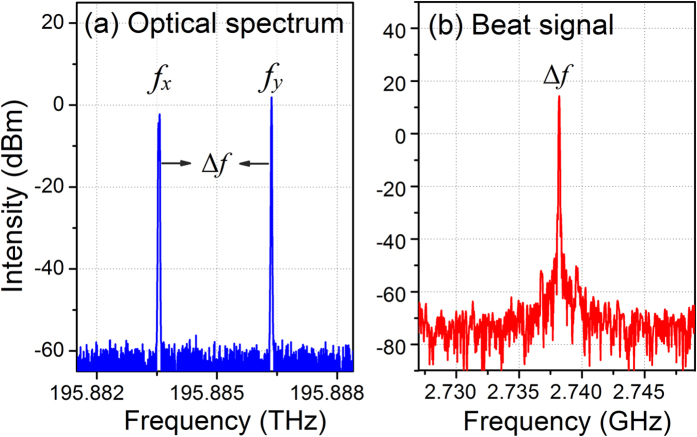
Measured optical spectrum (**a**) and the beat spectrum (**b**) of the laser output.

Figure 1(a) shows the schematic of ultrasound detection with a fiber laser. The laser (See Methods for details on the laser fabrication) naturally has two polarization modes with slightly different lasing frequencies *f*_x_ and *f*_y_, determined by the resonant condition^26^,^27^:


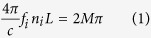


where *n*_*i*_ (*i* = *x* or *y*) denotes the effective refractive index of each mode, *c* is the speed of light in vacuum, *L* denotes the length of the laser cavity and *M* is an integer. These two orthogonal modes beat with each other at a radio frequency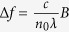, where *B* = |*n*_x_−*n*_y_| represents the intrinsic birefringence of the fiber. Here *n*_0_ represents the average effective refractive index for simplicity. An in-fiber polarizer is employed to maximize the ratio between beat signal and DC signal. The laser output is amplified by an Erbium-doped fiber amplifier (EDFA) Fig. 2(a) shows the spectrum of the output light measured with a high-resolution (10 MHz) optical spectrum analyzer (BOSA 200 CL, Aragon Photonics). The two polarization modes, lasing at 195.8835 and 195.8863 THz respectively, carry comparable output powers. The polarization hole-burning effect can significantly weaken the mode competition, which yields a stable lasing operation^28^. Figure 2(b) shows the output spectrum of the beat signal with a central frequency 2.738 GHz, corresponding to an intracavity birefringence of 2.05 × 10^−5^. This weak birefringence is mainly a result of the imperfection in geometrical control during fiber fabrication.

Figure 1(b) schematically shows the unperturbed and acoustically modulated beat signal, respectively. The perturbed beat signal can be demodulated using an I/Q data processing method. The configuration of the demodulation is shown in Fig. 1(a). The frequency-modulated signal is mixed with two low-noise quadrature radio frequency signals. The two radio frequency signals have an identical frequency close to that of the beating signal and have a 90-degree phase offset. After this quadrature down conversion, two baseband signals *I*(*t*) (In-phase) and *Q*(*t*) (quadrature) are used to depict the modulated signal. The phase information can be extracted via *φ*(*t*) = arctan(*Q*(*t*))⁄(*I*(*t*)). The frequency variation of the beat signal can be subsequently recovered by taking the derivative of the phase data.

**Figure 3:**
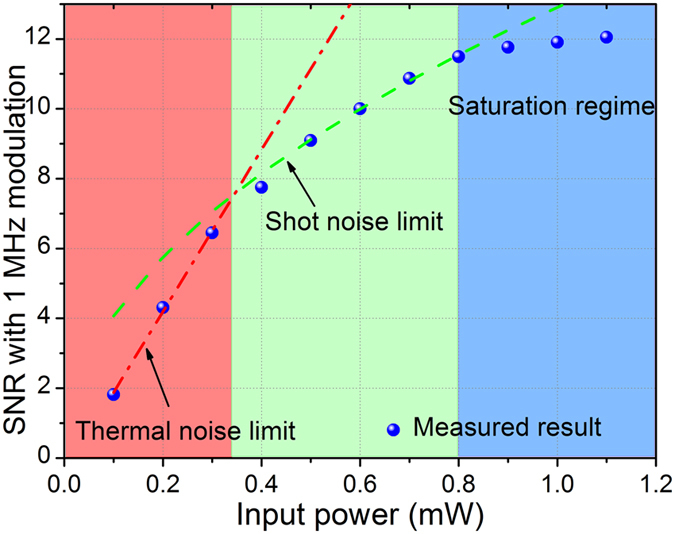
Measured signal-to-noise ratios (SNRs) as a function of input power at the photodetector. The amplitude of ultrasonically induced frequency shift was 1 MHz. The two dashed curves represent the inherent noise limits of the photodetector. The red and green regions represent the thermal-noise and shot-noise limited regimes. The blue region denotes the SNR is limited due to the saturation of the photodetector.

We tested the noise limits of the frequency demodulation with different optical power incident onto the photodetector, via gain adjustment of the fiber amplifier. Figure 3 shows the measured signal-to-noise ratios (SNRs) at different input optical power levels, with the same ultrasound excitation. The “signal” here refers to the ultrasound induced frequency variation and the “noise” is frequency fluctuation without ultrasound modulation. The ultrasound intensity was set at a level so that the maximum frequency shift was 1 MHz. The SNR increases with the input power and reaches around 12 when the power is amplified to 1.1 mW. The corresponding minimal detectable frequency shift is 83 kHz over 50 MHz bandwidths. The SNR is mainly limited by the noise figure of the photodetector. In low input power regime (less than 0.4 mW), the main limitation is thermal (Johnson) noise, which is independent of optical power. When the input power is greater than 0.4 mW, the SNR approximately depends on the square root of optical power, suggesting that shot noise becomes a dominant limiting factor. The photodetector becomes nearly saturated with input power over 1 mW. For broadband ultrasound signals, the noise-equivalent frequency shift (NEFS) of the frequency modulation system can be expressed by^29^


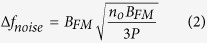


where *B*_*FM*_ denotes the bandwidths of interest, *n*_*o*_ represents the noise spectral density and *P* is the power of the beat signal, which is proportional to the square of optical power. For a fixed bandwidth, the NEFS is determined by the signal to noise ratio (SNR) of the beat signal 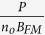.

The SNR of the beat signal is extremely high and the major limit lies on the performance of the photodetector and the dynamic range of the demodulation system. Because of common-mode noise cancellation between the two polarization modes, the beat signal has much lower phase noise than each polarization mode^30^, leading to a narrow linewidth of tens of Hertz, which corresponds to a detection limit of ~10^−11^ birefringence change. The ultrasound sensing resolution depends on the noise floor of the fiber laser, which is lower than −150 dBc/Hz. Considering the noise limitation of a high-power low-noise photodetector, we estimate the SNR can be improved about one order.

### Sensor characterization

**Figure 4:**
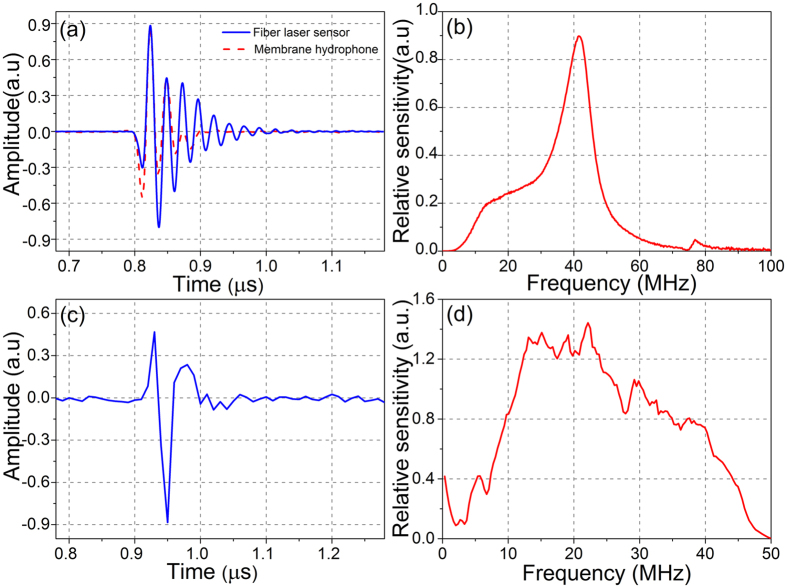
(**a**) Time response and (**b**) frequency response of a 65-μm fiber laser hydrophone to planar-wave ultrasound pulses. The measured time response by use of a commercial membrane hydrophone is superimposed for comparison^34^. (**c**) PA signal waveform recorded by the fiber laser hydrophone. (**d**) Frequency response to PA signals.

Fiber optic ultrasound detection can be treated as a problem of the scattering of the obliquely incident acoustic wave from a solid cylinder with infinite length^31^,^32^. The scattered wave establishes a non-uniform stress/strain profile over the optical fiber and induces changes in refractive index as well as fiber elongation. Assume the normally incident acoustic pressure is uniform over an infinite space, the induced frequency change can be expressed by 
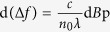
, where d*B*_p_ denotes the acoustically induced birefringence change, whose amplitude is determined by the decoupled wave equation as well as the displacement/stress continuity requirements at the solid-fluid interface. It has been found that the birefringence change scales with the product (*ε*_1_−*ε*_2_)•(*p*_11_−*p*_12_), where *ε*_1_ and *ε*_2_ are the principal strains at the fiber core, the *p*_11_ = 0.26 and *p*_12_ = 0.12 denote the Pockel’s constants which are related to the elastic properties of silica glass^33^. We first measured the temporal and frequency responses of the fiber laser ultrasound sensor under planar ultrasound wave excitation. Ultrasound pulses generated by an unfocused ultrasound transducer (V358-SU, Panametrics) were orthogonally exerted on the fiber laser. The front surface of the ultrasound transducer is 8 mm away from the fiber laser. The ultrasound source has been calibrated with a broadband hydrophone. Figure 4(a) shows the temporal response of a 65-μm fiber laser (the solid blue line). The vertical axis is the beat frequency shift. The laser has been etched by 40% hydrofluoric acid to reduce its diameter from 125 to 65 μm. The fiber laser sensor presents a frequency shift of 198 MHz in response to 88 kPa acoustic pressure, yielding a sensitivity of 2.25 MHz/kPa. Here the acoustic pressure is measured with a commercial hydrophone (HMB-0500, ONDA). The output signal is in good agreement with a commercial membrane hydrophone but shows some oscillations due to its limited bandwidth. The NEP of the detector is estimated as 40 Pa in 50 MHz bandwidth considering the noise floor at about 83 kHz. Figure 4(b) shows the fiber laser sensor’s frequency response. Figure 4(a) and (b) are obtained with a sampling rate up to several GHz order to give a full picture of its ultrasonic response. The fiber laser detector presents a sensitivity maximum at 41.6 MHz, which corresponds to the excitation of the 1^st^ radial and 2^nd^ circumferential mode. This mode is analogous to the case of periodically squeezing the fiber, which creates *ε*_1_ and *ε*_2_ with opposite signs. The considerable difference in Pockel’s constants offers an additive enhancement in the transduction of acoustic pressure to beat frequency change. The fiber is etched to eliminate possible excitation of higher-order vibration modes in the frequency range of interest (See Supple. Mat. for details on the response of a 125-μm fiber laser sensor).

**Figure 5:**
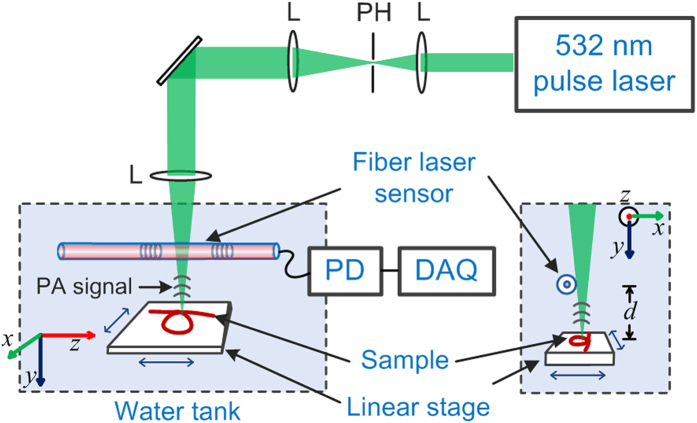
Experimental setup for the detection of PA signals. PH: Pinhole; L: Lens; PD: Photodetector; DAQ: Data acquirement system.

Figure 5 shows the experimental setup to measure photoacoustic (PA) signals with the fiber laser sensor. A 532-nm pulsed laser beam is focused on a piece of black tape via an objective lens (NA = 0.1). Pulse energy is ~100 nJ, and the pulse width is ~1.8 ns. The distance between the sample and the fiber laser *d* can be adjusted. Figure 4(c) and (d) exhibit a typical PA signal recorded by the fiber laser sensor and the corresponding frequency-domain signal. Based on the bandwidth of the real-time demodulation system, we set the sampling rate at 100 MHz, which allows a detection bandwidth of 50 MHz. The central frequency of the measured PA signal is ~23.2 MHz, and the bandwidth reaches over 32.5 MHz, which offers an axial resolution of 48 μm. The generated spherical PA wave can be approximated as a superposition of multiple planar waves with different incident angles. Each individual planar wave arrives at the line detector with a different phase. The interaction between the acoustic wave and the fiber can be treated as a uniform load over a finite effective length *L*_eff_ which approaches half acoustic wavelength, with the assumption of a nearly zero source-fiber distance *d*. The frequency response is modified as a result of the effective interaction length in inverse proportion to the ultrasound frequency.

**Figure 6:**
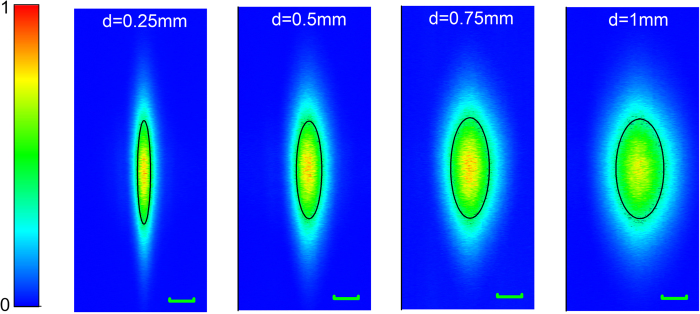
Measured in-plane field of view at different distances d. Scale bar: 0.5 mm.

Figure 6 shows the field of view at different distances *d* between the laser fiber sensor and the sample. We raster scanned the laser fiber sensor in the *x*-*y* plane over an area of 6.5 × 2.7 mm^2^ and keep others stationary. At each distance *d*, PA amplitudes over the scanned area are recorded to form an image. The sensitivity profile along the fiber direction remains ~2 mm with *d* varying from 0.25 to 1 mm. The sensitivity along the fiber is proportional to the intracavity light intensity at that specific position. The intracavity light intensity distribution is determined by the fiber gain, the coupling strength of the gratings as well as the grating separation^35^. The grating separation has been reduced as much as possible to yield high light intensity for sensitivity enhancement. The sensitivity profile normal to the fiber direction, in contrast, depends on the radial orientation of the fiber laser sensor in proportion to |cos(2*θ*)|, where *θ* represents the orientation angle with respect to one of the fiber principal axes. This cosine dependence arises from the core asymmetry, which can be considered as a first-order perturbation to a perfect circle. As a result, the ultrasonic response reaches its maximum at fiber principal axis directions. The full angle at half maximum is 60 degrees. As a result, the effective field of view normal to the fiber direction scales with the distance *d*. The measured effective length at *d* = 1 mm is 0.98 mm, in accordance with this prediction. The field of view (FOV) presents an effective area of 1.57 mm^2^ at *d* = 1 mm with a nearly elliptical profile. Fiber laser sensor works as both line and sideway probe. This is favorable in clinical endoscopes and intravascular application. This large FOV benefit many applications. Most other optical acoustic sensors are not focused; therefore one need to put the sensor close to the sample to receive more acoustic energy, leading to very limited FOV. Since our fiber laser sensor has a constant sensitivity along the fiber, it can keep large FOV while being placed close to the sample.

**Figure 7:**
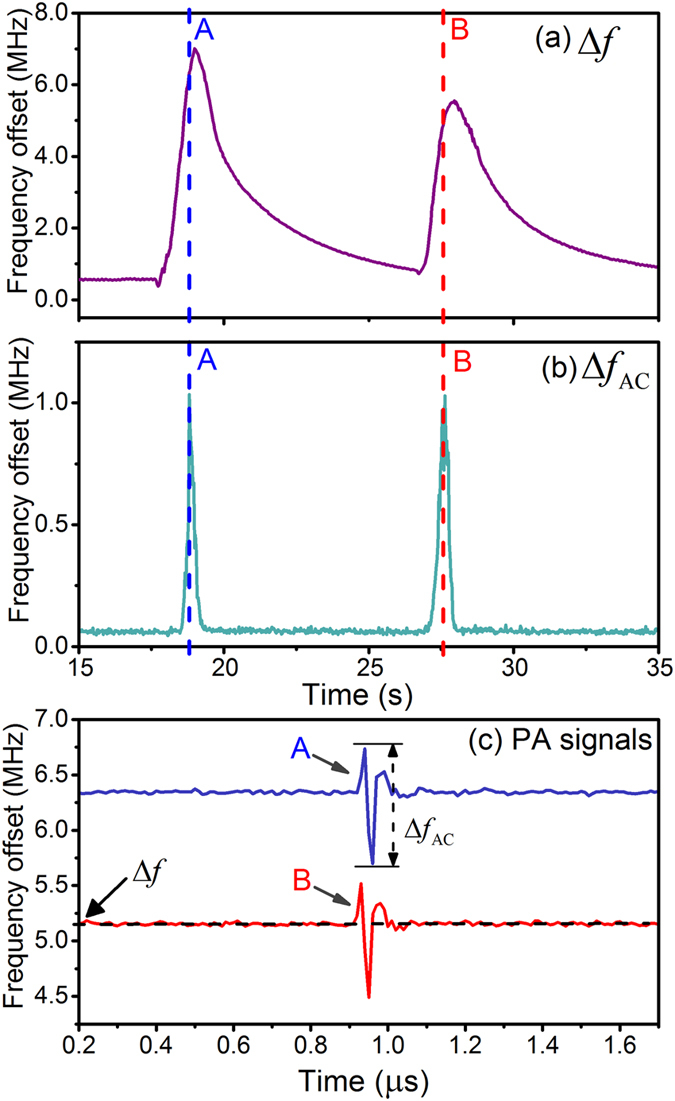
(**a**) Measured bending induced slowly-varying beat-frequency variation (denoted as Δf) and (**b**) recorded peak-to-peak amplitudes of PA waveforms (denoted as Δf_AC_) during a fast scanning process. (**c**) Measured PA waveforms at the moments A and B which correspond to the positions with maximum responses for two individual scanning cycles. The measurement of PA signal is not affected by the applied disturbance.

To test the stability of the fiber laser sensor, we scanned the sensor along the cross-fiber direction back and forth to generate a disturbance. The fiber laser sensor is held at one end and translated with a linear stage. The maximum scanning speed reaches 10 mm/s. During the scanning process, the beat frequency changes as a result of bending-induced birefringence change. Figure 7(a) shows this slow frequency variation Δ*f* of the “carrier signal”. Figure 7(c) shows recorded PA waveforms at the moments A and B which correspond to the positions with maximum responses for two individual scanning cycles. It suggests that the PA waveforms can be extracted with high repeatability despite the strong disturbance. Figure 7(b) plots the variation in amplitude Δ*f*_AC_ of the recorded PA waveforms, which represents the sensitivity profile along the cross-fiber direction. The fiber laser naturally operates in resonance mode, which avoids the need for wavelength locking. The high stability of the beat-frequency encoded fiber laser sensor is attributed to the self-heterodyning manner. Unlike many other fiber laser sensors which measure the phase shift of each polarization mode, our fiber laser sensor directly sense the phase difference between the two orthogonal modes, thus the common-mode perturbations such as temperature changes and vibrations are canceled between the two modes.

### Photoacoustic Microscopy

**Figure 8:**
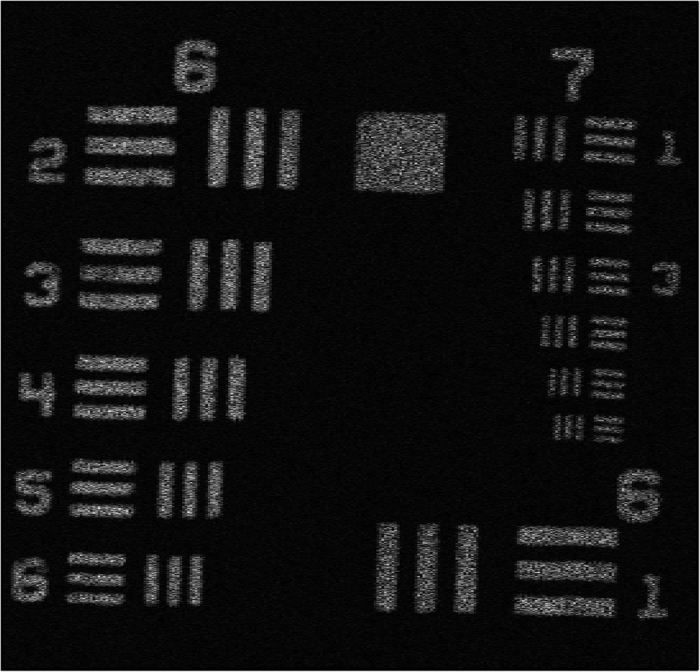
OR-PAM image of a resolution test target obtained by using a fiber laser ultrasound sensor.

Optical-resolution photoacoustic microscopy (OR-PAM) has been demonstrated with the fiber laser ultrasound sensor, based on the configuration shown in Fig. 5. The 532 nm light pulses are incident along the *y*-axis to excite PA signals. The PAM works in a reflection mode by fixing the fiber laser ultrasound sensor above the sample. The fiber laser sensor is placed slightly off the optical path to avoid scattering of the excitation light and affect the spatial resolution. To maximize the sensitivity, the fiber laser sensor is rotated around fiber axis, so that we can align the detection principle axis of the sensor with the propagation direction of the ultrasound wave. We raster scanned the sample in the *x*-*z* plane while keeping other components stationary. Figure 8 shows the photoacoustic image of a resolution test target (R1DS1P, Thorlabs) for lateral resolution test. The sixth elements in group 7 were clearly resolved. The Modulation transfer function (MTF) for this element group is about 20% and the lateral resolution is estimated as 3.2 μm. This value is close to the theoretical diffraction-limited resolution at 532 nm with NA = 0.1. The result indicates that the use of this sensor can achieve an optical diffraction-limited imaging resolution.

**Figure 9:**
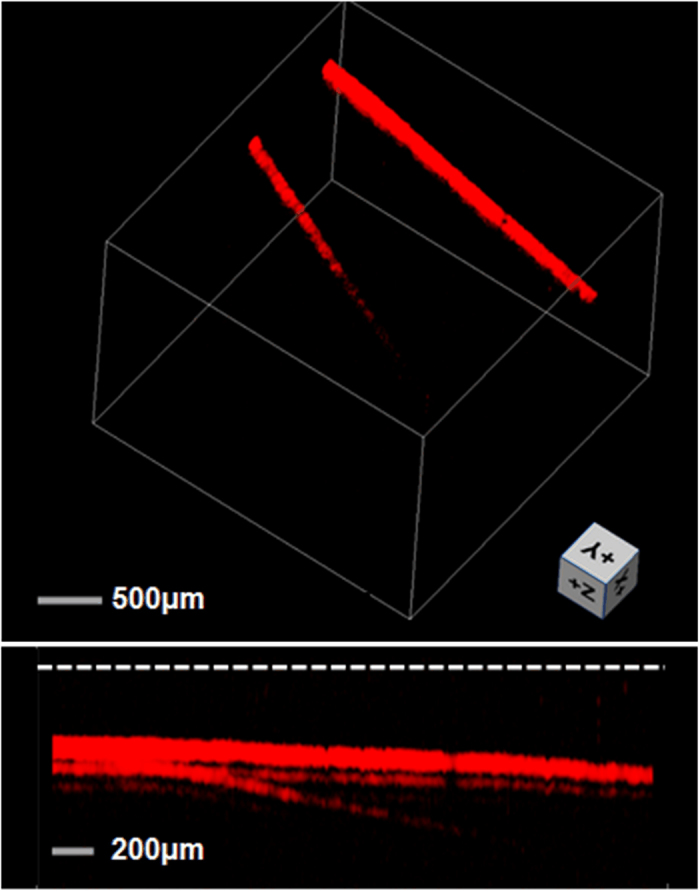
Test of penetration depth by imaging two human hairs inserted into the biological tissue. The result is presented in volumetric (upper) and side (lower) views, respectively. The white dashed line in the lower image represents the tissue surface.

**Figure 10:**
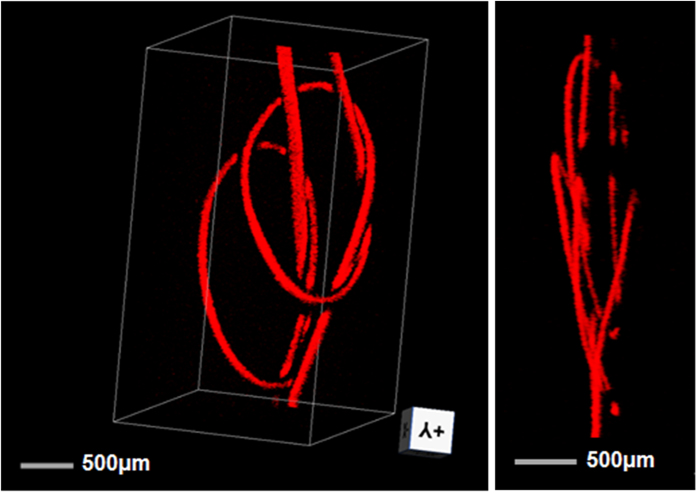
Volumetric PAM image of two hair knots in different angles of view.

The penetration depth of the PAM was measured by imaging two human hairs which are obliquely inserted into a piece of fresh chicken breast. The focus depth is about 400 μm. The light intensity at the tissue surface was ~20 mJ/cm^2^, within the ANSI safety limit. Figure 9 shows the volumetric and side views of the image (See Supple. movie 1). The hairs are visible at a depth beyond 0.8 mm in biological tissue. Figure 10 shows the volumetric PAM image of two human hairs in knot structure. The image is presented with two different view angles, showing that the 3-D knot structure can be clearly resolved (See Supple. movie 2). The hair images in Fig. 9 are acquired with a 125-μm-diameter sensor for the detection of PA signals, which shows shadowing effect as a result of the narrow bandwidth around 40 MHz (See Supple. Fig. S1). This 40 MHz peak in frequency response is a result of effective excitation of the 2nd radial and 2nd azimuthal mechanical mode. This peak can be shifted out of the working bandwidth by reducing fiber diameter. In comparison, the images in Fig. 10 acquired with the 65-μm-diameter fiber laser sensor, presents a better visibility than that in Fig. 9, due to the improved frequency response.

## Conclusion

In summary, we demonstrate ultrasound detection with a short cavity fiber laser for photoacoustic microscopy. The fiber laser sensor presents great advantages including high sensitivity, low NEP, broad bandwidth, miniature size and inherent stability. A NEP of 40 Pa over a 50-MHz bandwidth has been achieved with a 65-μm fiber laser. The NEP can be improved by using a photodetector with higher saturation power to further enhance the SNR with higher optical power. Taking advantage of common-mode cancellation, the output beat signal frequency presents inherent resistance to external perturbations, without the use of any frequency-locking techniques. Preliminary results of photoacoustic imaging with the fiber laser sensor have been demonstrated. OR-PAM with axial and lateral resolutions of 48 and 3.3 μm have been achieved. The sensor offers a side-looking FOV of up to 1.57 mm^2^. The fiber laser sensor provides a new tool for developing new photoacoustic imaging modalities. The miniature size, as well as the side-looking manner, enables future endoscopic applications. Nevertheless, a large amount of work is needed towards the realization of a fiber-laser-sensor based PA endoscopy.

## Methods

### Fabrication of the fiber laser

The fiber laser is fabricated by imprinting two wavelengths matched fiber Bragg gratings (FBGs) in series in an Er-Yb codoped fiber (EY-305, Fibercore). An FBG refers to intra-core periodic index modulation, formed by illuminating the fiber by use of a 193 nm excimer laser through the diffraction of a phase mask. Each grating acts as a distributed reflector with a coupling strength κ above 10 cm^−1^ at the Bragg wavelength, enabling strong optical feedbacks for laser oscillation. The grating pitch is Λ = 1053 nm and the corresponding reflective wavelength is *λ*_B_ = *n*_eff_Λ = 1532 nm, where *n*_eff_ denotes the effective index of the fiber. This wavelength is located at around the absorption peak of the rare-earth doped fiber to maximum the intracavity gain. The gratings are 6 and 5 mm in length, respectively. The gratings have a separation of less than 0.5 mm. The output power of such a laser is typically 500 μW, with a pump power of 100 mW at 980 nm.

## Additional Information

**How to cite this article**: Liang, Y. *et al*. Fiber-Laser-Based Ultrasound Sensor for Photoacoustic Imaging. *Sci. Rep.* **7**, 40849; doi: 10.1038/srep40849 (2017).

**Publisher's note:** Springer Nature remains neutral with regard to jurisdictional claims in published maps and institutional affiliations.

## Supplementary Material

Supplementary Information

Supplementary Video 1

Supplementary Video 2
