# Supplementary material for: Fiber-Laser-Based Ultrasound Sensor for Photoacoustic Imaging
Source: Sci Rep. 2017 Jan 18;7:40849. doi: 10.1038/srep40849 (PMC5241646; doi:10.1038/srep40849)
Supplement: Supplementary Information [file srep40849-s1.pdf]

Supplementary information for:

Fiber Laser Ultrasound Sensor for Photoacoustic Imaging

Yizhi Liang[1, 2], Long Jin*[1], Lidai Wang*[2], Xue Bai[1], Linghao Cheng[1], and Bai-Ou Guan[1]

[1]*Guangdong Provincial Key Laboratory of Optical Fiber Sensing and Communications, Institute of Photonics Technology, Jinan University, Guangzhou 510632, China*

[2]*Department of Mechanical and Biomedical Engineering, City University of Hong Kong, Kowloon Tong, Hong Kong*

* Corresponding to: iptjinlong@gmail.com, lidawang@cityu.edu.hk

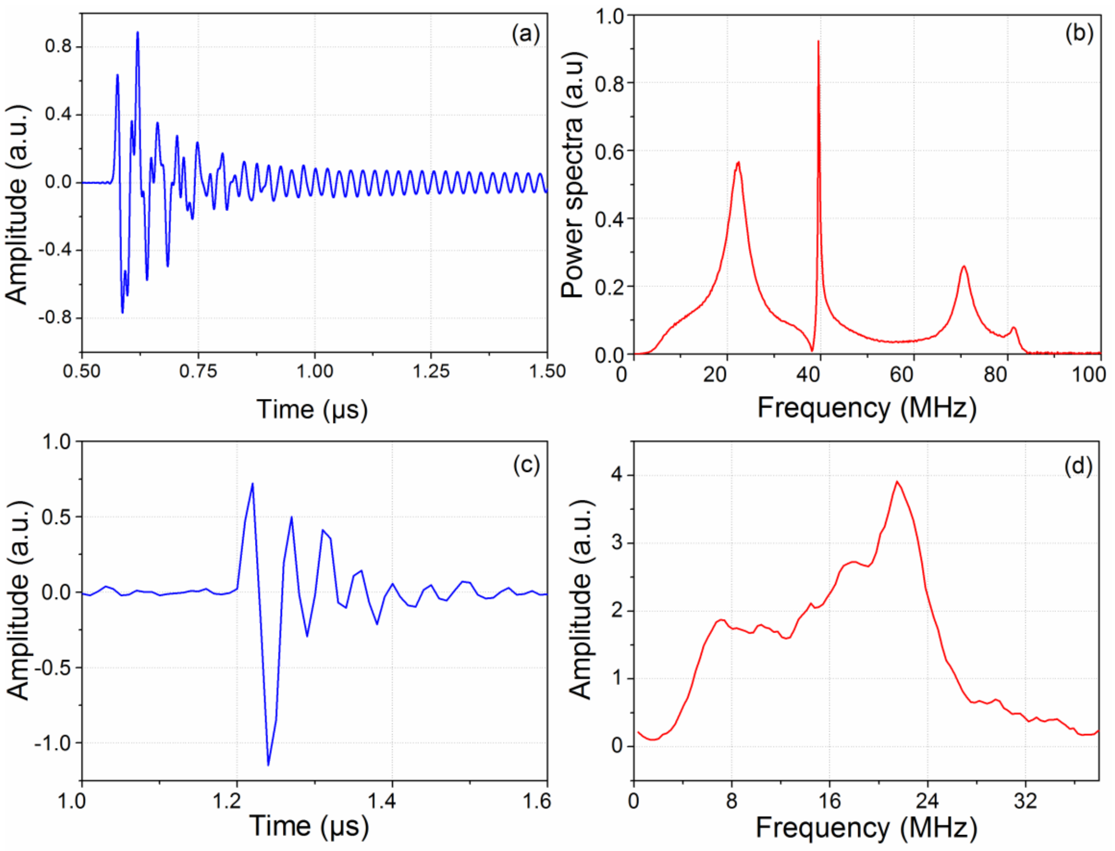

Supplementary Figure S1: (a) Time response and (b) frequency response of a 125-μm fiber laser sensor to planar-wave ultrasound pulses. (c) PA signal waveform recorded by the 125-μm fiber laser sensor. (d) Frequency response to PA signals.

Supplementary videos:

Video S1: Volumetric PAM image of two human hairs inserted into the biological tissue.

Video S2: Volumetric PAM image of two hair knots.
